# Supplementary material for: Clinical and electrophysiological features of SCN8A variants causing episodic or chronic ataxia
Source: eBioMedicine. 2023 Oct 28;98:104855. doi: 10.1016/j.ebiom.2023.104855 (PMC10628346; doi:10.1016/j.ebiom.2023.104855)
Supplement: Supplementary Figures, Tables and Video legends [file mmc1.docx]

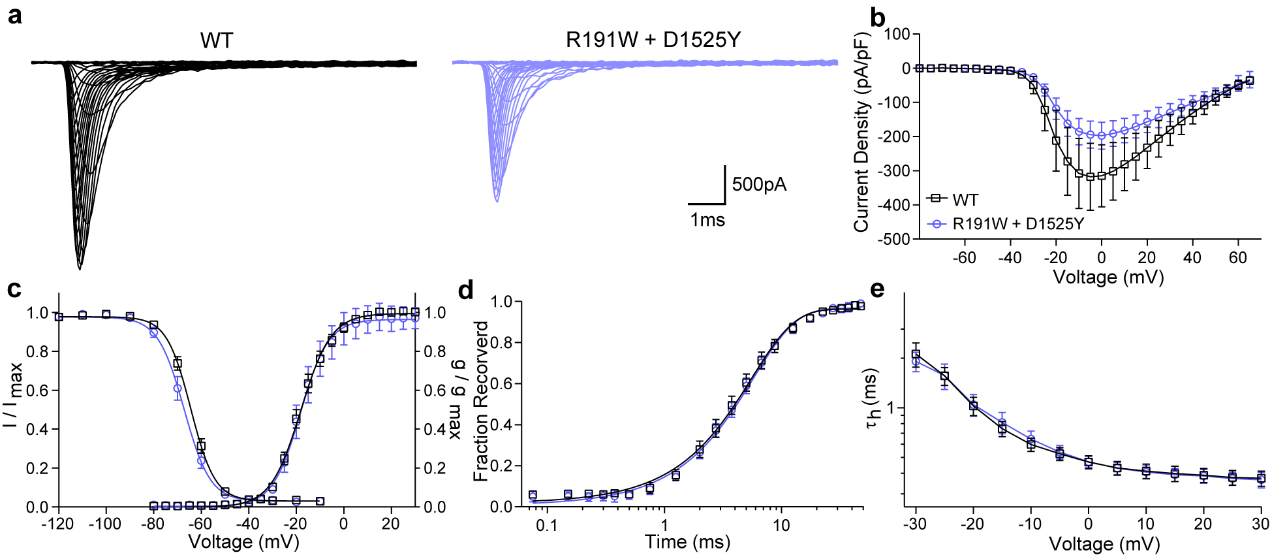


**Supplementary Figure 1: Functional studies of biallelic variants R191W and D1525Y in ND7/23 cells.** (a) Representative traces of Na^+^ current for WT and R191W+D1525Y (co-expression with the ratio of 1:1). (b) Peak Na^+^ currents normalised by cell capacitances were plotted versus voltage. (c) Voltage-dependent steady state activation and inactivation curves. Lines represent Boltzmann functions fit to the data points. (d) Time course of recovery from fast inactivation at -100 mV. (e) Voltage-dependence of the time constant of fast inactivation τ_h_. All data are shown as means ± 95% confidence interval. Numbers of recorded cells and statistical analysis are provided in Supplementary Table 2.

**
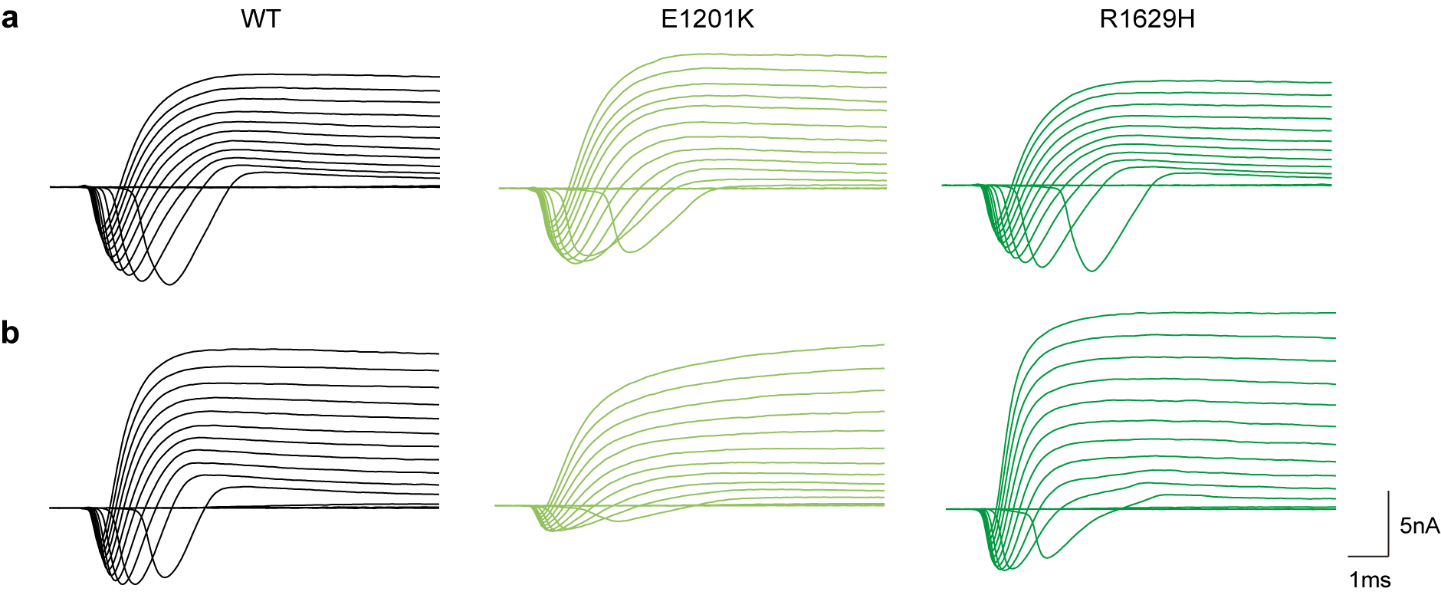
**

**Supplementary Figure 2: Representative traces of Na^+^ current recorded in transfected hippocampal neuronal cultures in presence of TTX.** (a) Representative Na^+^ current traces recorded in transfected excitatory neurons. (b) Representative sodium current traces recorded in transfected inhibitory neurons. E1201K shows a decreased Na^+^ current amplitude compared with WT and R1629H.

**
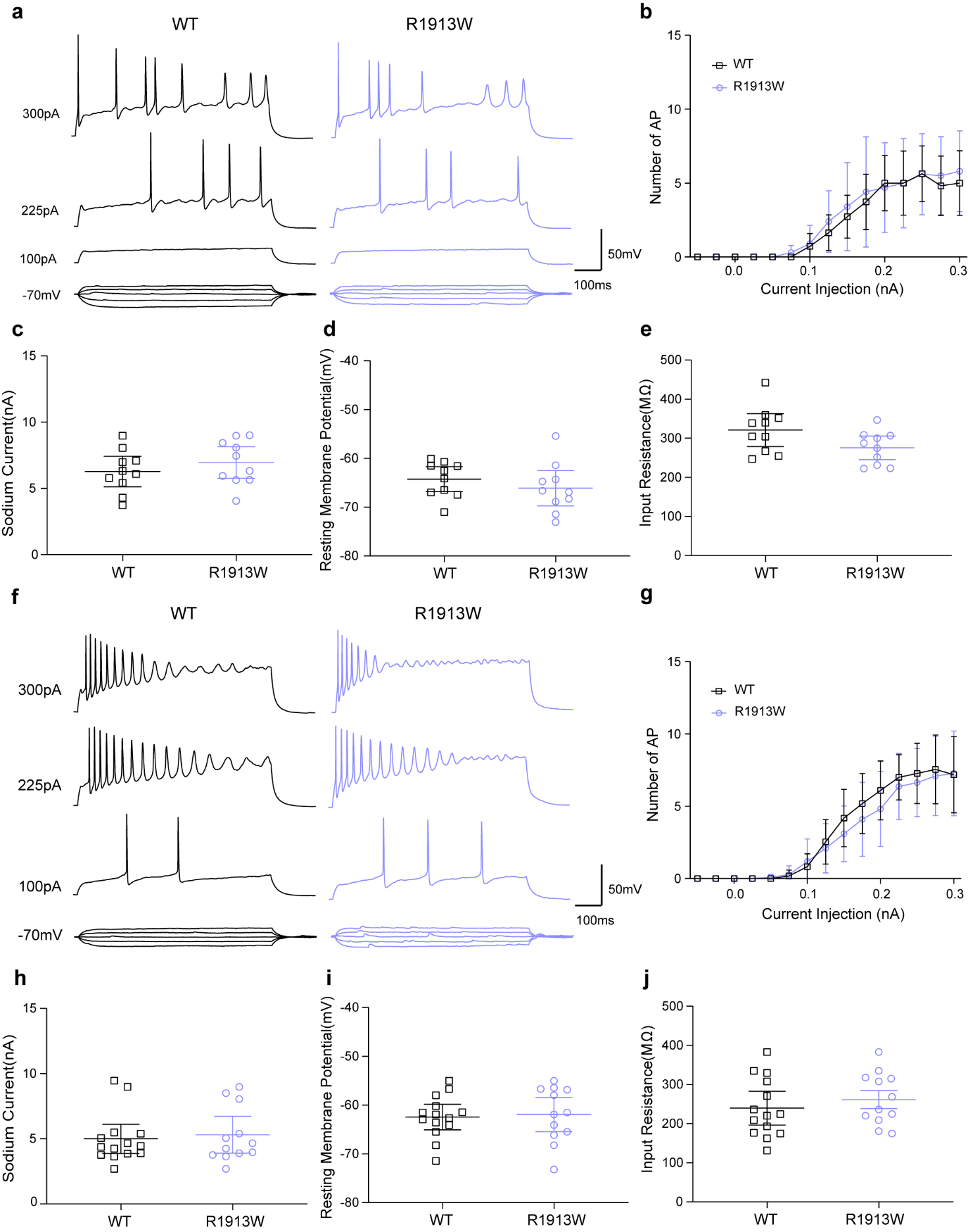
**

**Supplementary Figure 3: Intrinsic neuronal and firing properties of cultured hippocampal excitatory and inhibitory neurons transfected with *SCN8A* WT or R1913W in presence of TTX.** (a) Representative firing traces of evoked action potentials (APs) recorded in hippocampal excitatory neurons transfected with WT and R1913W. (b) Number of APs plotted versus injected current. WT, *n* = 11; R1913W, *n* = 10. (c) Peak Na^+^ current amplitudes, (d) Resting membrane potential and (e) Input resistance of transfected excitatory neurons. (f) Representative firing traces of evoked APs recorded in hippocampal inhibitory neurons transfected with WT and R1913W. (g) Number of APs plotted versus injected current. WT, *n* = 14; R1913W, *n* = 12. (h) Peak Na^+^ current amplitudes, (i) Resting membrane potential and (j) Input resistance of transfected excitatory neurons. All data are presented as means ± 95% confidence interval. Detailed statistical analysis is provided in Supplementary Table 3.

**Supplementary Table 1. Clinical and demographic features of the study participants.** Abbreviations: NA – not available/applicable; ND – not done; AZA – acetazolamide; VPA – valproic acid; LTG –lamotrigine; OXC – oxcarbazepine; CBZ – carbamazepine; CLB – clobazam; LEV – levetiracetam; SEP – somatosensory evoked potentials; STP – stiripentol; VEP – visual evoked potentials; VPA – valproic acid.

|  | Steady-state activation | | | Steady-state inactivation | | | Trec at -100 mV, ms | n | Th at 0 mV, ms | Persistent current,  % of peak current | Current density, pA/pF | n |
| --- | --- | --- | --- | --- | --- | --- | --- | --- | --- | --- | --- | --- |
|  | V ½, mV | k | n | V ½, mV | k | n |  |  |  |  |  |  |
| WT | -18.0 ± 4.1 | -5.3 ± 0.7 | 19 | -64.2 ± 1.7 | 4.9 ± 0.53 | 19 | 5.2 ± 0.9 | 19 | 0.47 ± 0.07 | 0.33 ± 0.41 | -319.3 ± 163.7 | 19 |
| R191W + D1525Y | -17.6 ± 4.2 | -5.5 ± 1.4 | 17 | -67.3 ± 2.8  (*P* = 0.028) | 5.3 ± 0.40 | 17 | 5.1 ± 0.9 | 17 | 0.46 ± 0.09 | 0.57 ± 0.49 | -199.6 ± 79.2 | 17 |

**Supplementary Table 2. Biophysical properties of *SCN8A* WT and R191W+D1525Y recorded in ND7/23 cell.** Data are presented as means ± SD; *n* = number of recorded cells; WT = wild-type.

|  | Sodium current, pA | Resting membrane potential (RMP), mV | Input resistance, MΩ | n |
| --- | --- | --- | --- | --- |
| WT - excitatory | 6276 ± 1602 | -64.3 ± 3.6 | 321 ± 59 | 11 |
| R1913W - excitatory | 6959 ± 1677 | -66.1 ± 5.1 | 275 ± 42 | 10 |
| WT - inhibitory | 4998 ± 1936 | -70.4 ± 4.5 | 240 ± 75 | 14 |
| R1913W - inhibitory | 5110 ± 2235 | -71.6 ± 5.5 | 261 ± 79 | 12 |

**Supplementary Table 3. Intrinsic neuronal properties in transfected excitatory or inhibitory hippocampal neurons in the presence of TTX.** Data are presented as means ± SD; *n* = number of recorded cells; WT = wild-type.

**Supplementary Video Legends**

**Supplementary. Video 1. Phenotype of individual 3.** Age at examination: 13 years of age. A. She has always had difficulty running normally. In the video, there is minimal knee flexion and genu valgum while running. B. She climbs the stairs more slowly than normal, despite the fact that she does not require assistance. C. The nose-finger and finger-finger tests are perfectly normal, and she does not have tremor. D. She presents slight difficulties in her gait, only visible when she walks in tandem.

**Supplementary. Video 2. Phenotype of individual 6.** A. At 2 months of age, she presented a conjugated pendular slow vertical nystagmus. Nystagmus was present most of the time. B. Conjugated pendular vertical nystagmus may be noticed in this video during one of the ataxia episodes caused by febrile infection. C. Slow pendular horizontal nystagmus during another episode of acute ataxia. D, E, F. When able to walk, she was noted to be able to stand in a natural position with a sway without assistance. She also stumbled somewhat when she started walking. G. The video depicts her present condition between episodes without ataxia and the ability to climb obstacles without difficulty.
