# Supplementary material for: Clinical and electrophysiological features of SCN8A variants causing episodic or chronic ataxia
Source: eBioMedicine. 2023 Oct 28;98:104855. doi: 10.1016/j.ebiom.2023.104855 (PMC10628346; doi:10.1016/j.ebiom.2023.104855)
Supplement: Antibody validation [file mmc3.pdf]

GAD67 Monoclonal Antibody (OTI3G9)

**Product Details**	
Size	100 µL
Species Reactivity	Human, Mouse, Non-human primate, Rat
Published Species	Rhesus monkey
Host/Isotype	Mouse / IgG2b
Class	Monoclonal
Type	Antibody
Clone	OTI3G9
Conjugate	Unconjugated
Immunogen	Full length human recombinant protein of GAD1 produced in HEK293T cell
Form	Liquid
Concentration	1 mg/mL
Purification	Affinity Chromatography
Storage buffer	PBS, pH 7.3, with 1% BSA, 50% glycerol
Contains	0.02% sodium azide
Storage conditions	-20° C, Avoid Freeze/Thaw Cycles
RRID	AB_2723202

**Applications**	**Tested Dilution**	**Publications**
Western Blot (WB)	1:1,000-1:2,000	1 Publication
Immunohistochemistry (IHC)	-	1 Publication
Immunohistochemistry (Paraffin) (IHC (P))	1:50	-
Immunocytochemistry (ICC/IF)	1:50	1 Publication
Immunoprecipitation (IP)	4 µg/mL	-

Product Images For GAD67 Monoclonal Antibody (OTI3G9)

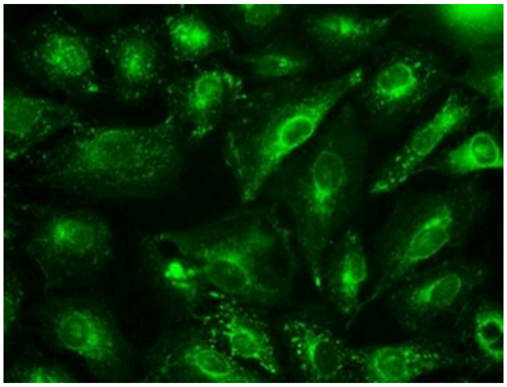

GAD67 Antibody (MA5-24909) in ICC/IF
Immunofluorescent analysis of GAD1 in A549 cells. Cells were probed with a GAD1 monoclonal antibody (Product # MA5-24909).

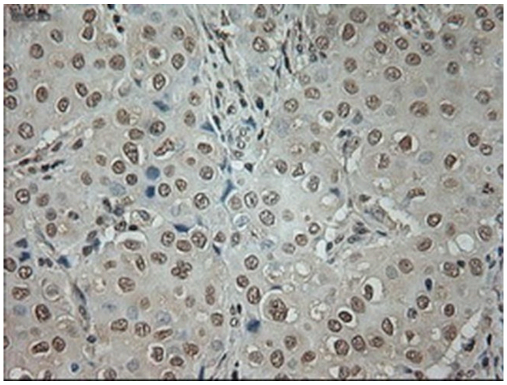

GAD67 Antibody (MA5-24909) in IHC (P)
Immunohistochemistry was performed on paraffin-embedded adenocarcinoma of human breast tissue. To expose target proteins, 10mM citric buffer, pH6.0, 100°C for 10min was used. Following antigen retrieval, tissues were probed with a GAD1 monoclonal antibody (Product # MA5-24909).

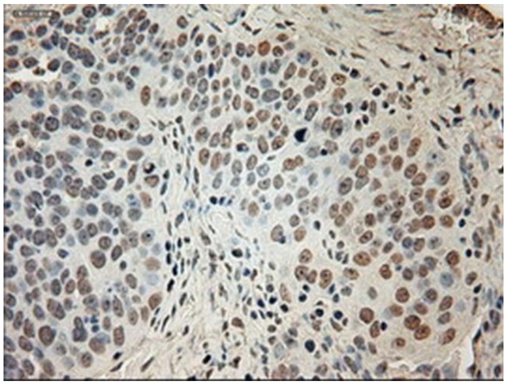

GAD67 Antibody (MA5-24909) in IHC (P)
Immunohistochemistry was performed on paraffin-embedded adenocarcinoma of human ovary tissue. To expose target proteins, 10mM citric buffer, pH6.0, 100°C for 10min was used. Following antigen retrieval, tissues were probed with a GAD1 monoclonal antibody (Product # MA5-24909).

View more figures on thermofisher.com

3 References

Western Blot (1)

CNS neuroscience & therapeutics

Deep brain stimulation of the anterior nuclei of the thalamus relieves basal ganglia dysfunction in monkeys with temporal lobe epilepsy.

"MA5-24909 was used in Western Blotting to study the effects of anterior nuclei of the thalamus on the basal ganglia in monkeys."

Authors: Du T,Chen Y,Shi L,Liu D,Liu Y,Yuan T,Zhang X,Zhu G,Zhang J

Year
2021

Species
Rhesus monkey

Dilution
1:1000

Immunohistochemistry (1)

CNS neuroscience & therapeutics

Deep brain stimulation of the anterior nuclei of the thalamus relieves basal ganglia dysfunction in monkeys with temporal lobe epilepsy.

"MA5-24909 was used in Western Blotting to study the effects of anterior nuclei of the thalamus on the basal ganglia in monkeys."

Authors: Du T,Chen Y,Shi L,Liu D,Liu Y,Yuan T,Zhang X,Zhu G,Zhang J

Year
2021

Species
Rhesus monkey

Dilution
1:1000

Immunocytochemistry (1)

CNS neuroscience & therapeutics

Deep brain stimulation of the anterior nuclei of the thalamus relieves basal ganglia dysfunction in monkeys with temporal lobe epilepsy.

"MA5-24909 was used in Western Blotting to study the effects of anterior nuclei of the thalamus on the basal ganglia in monkeys."

Authors: Du T,Chen Y,Shi L,Liu D,Liu Y,Yuan T,Zhang X,Zhu G,Zhang J

Year
2021

Species
Rhesus monkey

Dilution
1:1000

For Research Use Only. Not for use in diagnostic procedures. Not for resale without express authorization. Products are warranted to operate or perform substantially in conformance with published Product specifications in effect at the time of sale, as set forth in the Production documentation, specifications and/or accompanying package inserts ("Documentation"). No claim of suitability for use in applications regulated by FDA is made. The warranty provided herein is valid only when used by properly trained individuals. Unless otherwise stated in the Documentation, this warranty is limited to one year from date of shipment when the Product is subjected to normal, proper and intended usage. This warranty does not extend to anyone other than the Buyer. Any model or sample furnished to Buyer is merely illustrative of the general type and quality of goods and does not represent that any Product will conform to such model or sample. NO OTHER WARRANTIES, EXPRESS OR IMPLED, ARE GRANTED INCLUDING WITHOUT LIMITATION, IMPLIED WARRANTIES OF MERCHANTABILITY, FITNESS FOR ANY PARTICULAR PURPOSE, OR NON INFRINGEMENT. BUYER'S EXCLUSIVE REMEDY FOR NON-CONFORMING PRODUCTS DURING THE WARRANTY PERIOD IS LIMITED TO REPAIR, REPLACEMENT OF OR REFUND FOR THE NON-CONFORMING PRODUCT(S) AT SELLER'S SOLE OPTION. THERE IS NO OBLIGATION TO REPAIR, REPLACE OR REFUND FOR PRODUCTS AS THE RESULT OF (I) ACCIDENT, DISASTER OR EVENT OF FORCE MAJEURE, (II) MISUSE, FAULT OR NEGLIGENCE OF OR BY BUYER, (III) USE OF THE PRODUCTS IN A MANNER FOR WHICH THEY WERE NOT DESIGNED, OR (IV) IMPROPER STORAGE AND HANDLING OF THE PRODUCTS. Unless otherwise expressly stated on the Product or in the documentation accompanying the Product, the Product is intended for research only and is not to be used for any other purpose, including without limitation, unauthorized commercial uses, in vitro diagnostic uses, ex vivo or in vivo therapeutic uses, or any type of consumption by or application to human or animals.
